# Statins use is associated with poorer glycaemic control in a cohort of hypertensive patients with diabetes and without diabetes

**DOI:** 10.1186/1758-5996-6-53

**Published:** 2014-04-23

**Authors:** Su May Liew, Ping Yein Lee, Nik Sherina Hanafi, Chirk Jenn Ng, Stalia Siew Lee Wong, Yook Chin Chia, Pauline Siew Mei Lai, Nur Farhana Mohd Zaidi, Ee Ming Khoo

**Affiliations:** 1 Department of Primary Care Medicine, University of Malaya Primary Care Research Group (UMPCRG), Faculty of Medicine, University of Malaya, Kuala Lumpur, Malaysia; 2 Department of Family Medicine, Universiti Putra Malaysia, Serdang, Selangor, Malaysia

**Keywords:** Hypertension, Diabetes mellitus, Statin, HbA1c

## Abstract

**Background:**

The US Federal and Drug Administration (FDA) recently revised statin drug labels to include the information that increases in fasting serum glucose and glycated haemoglobin levels have been reported with the use of statins. Yet in a survey, 87% of the doctors stated that they had never or infrequently observed increases in glucose or HbA1c levels in patients on statin. In this study we would like to determine the association between the use of statins and glycaemic control in a retrospective cohort of patients with hypertension.

**Methods:**

A retrospective review of 1060 medical records of patients with hypertension at a primary care clinic was conducted. These records were selected using systematic random sampling (1:4). Data on patient socio-demographic factors; clinical profile; investigation results and prescribed medications were collected. Independent *t*-test was used for continuous variables while Pearson’s *χ*2 test was used for categorical variables. Logistic regression was done to adjust for confounders.

**Results:**

810 (76.4%) patients with hypertension were on statins, out of which 792 (97.8%) were taking simvastatin 10 mg or 20 mg daily. Analysis of the whole group regardless of diabetes status showed that the statin user group had higher HbA1c and fasting blood glucose values. The difference in HbA1c levels remained significant (adjusted OR = 1.290, p = 0.044, 95% CI 1.006, 1.654) after adjustment for diabetes, diabetic medication and fasting blood glucose. In the study population who had diabetes, statin users again had significantly higher HbA1c level compared to statin non-users. This difference remained significant (adjusted OR 1.208, p = 0.037, 95% CI 1.012, 1.441) after adjustment for age and diabetic medications.

**Conclusions:**

Statins use is associated with increased HbA1c levels among hypertensive patients and hypertensive patients with diabetes. Clinicians managing hypertensive patients on statins should consider monitoring the HbA1c level and ensure that those with diabetes have their hyperglycaemia kept under control.

## Background

In February 2012, the US Federal and Drug Administration (FDA) revised statin drug labels to include the information that increases in fasting serum glucose and glycated haemoglobin levels have been reported with the use of statins [1]. The changes were based on evidence from two trials [2,3] as well as meta-analysis and cohort studies [4-6]. Statin therapy was found to be associated with an increase in the incidence of diabetes and worsening glycaemic control. This association is worrying because diabetes doubles the risk of cardiovascular disease and cardiovascular deaths account for about 50% of deaths in people with diabetes [7]. Poor glycaemic control also increases the risk of cardiovascular outcomes. The United Kingdom Prospective Diabetes Study (UKPDS) trial showed that reduction in glycated haemoglobin by 1% was associated with a 21% decrease in the risk of any end point or death related to diabetes [8].

Studies have also shown that statins worsened glycaemic control in patients with diabetes [9,10]. Yet in a statin survey, 87% of the doctors stated that they had never or infrequently observed increases in glucose or HbA1c levels in patients on statin [11]. This study aimed to determine the association between the use of statins and glycaemic control in a retrospective cohort of patients with hypertension.

## Methods

A retrospective review of medical records was conducted between January and May 2012 at a primary care clinic in a tertiary teaching hospital in Malaysia. Inclusion criteria were 1) patient with hypertension above the age of 18 years, 2) patient had been followed up for at least one year for hypertension at the clinic, and 3) patient who attended follow-up appointment within the study period. Hypertension was defined as a diagnosis of hypertension as stated in medical records or was on antihypertensive medication. Approximately 4,000 patients with hypertension attended their follow-up appointments during the study period. Records were selected using the systematic random sampling (1:4) method, giving a total of 1060 records.

An electronic self-designed data collection form was used to collect the following data: patient socio-demographic factors; clinical profile; investigation results and prescribed medications. Data were collected by two trained research assistants. The most recent medication list and investigation results were obtained from patient records and verified with the hospital’s electronic record system. Diabetes mellitus was defined as a documentation of the diagnosis in the medical record or patients with a fasting blood glucose of 7 mmol/L and above or those who were prescribed medication for diabetes.

### Data analyses

All data was analysed using the Statistical Package for Social Sciences (SPSS) version 18 (Chicago, Illinois, USA). Continuous data were expressed as mean ± standard deviation (SD). Categorical variables were expressed as absolute (number) and relative frequencies (percentage). Independent *t*-test was used for comparison of continuous variables while Pearson’s *χ*2 test was used for comparison of categorical variables.

All factors that were significantly associated with statin use in the bivariate analysis, or contributed to the level of diabetic control, were entered into a logistic regression model. A p-value of <0.05 was considered statistically significant.

### Ethical approval

Ethical approval for this study was obtained from the University of Malaya Medical Ethics Committee (Reference number: 890.14). Data were anonymised and patient confidentiality was maintained.

## Results

Table 1 shows the types of statins and dosages prescribed in our study population: 810 (76.4%) patients with hypertension were on statins, out of which 792 (97.8%) were taking simvastatin 10 mg or 20 mg daily.

**Table 1:** Types of statins and dosages prescribed in study population

**Type of statin prescribed (n = 810)**	**Dosage (mg)**	**n (%)**
Simvastatin (n = 792)	5.0	1 (0.1)
	10.0	239 (30.2)
	20.0	440 (55.6)
	30.0	11 (1.4)
	40.0	99 (12.6)
	80.0	1 (0.1)
Atorvastatin (n = 12)	10.0	3 (25.0)
	20.0	4 (33.3)
	30.0	1 (8.3)
	40.0	4 (33.3)
Pravastatin (n = 4)	20.0	3 (75.0)
	40.0	1 (25.0)
Rosuvastatin (n = 2)	10.0	1 (50.0)
	20.0	1 (50.0)

Table 2 shows the results of the entire study population regardless of their diabetes status. A significantly greater number of hypertensive patients who used statins were diabetics (51.2% vs. 36.8%) and were on diabetic medications (48.3% vs. 30.4%) compared with non-users. Statin users had higher HbA1c and fasting blood glucose (FBG) values, compared to non-users. The difference in HbA1c levels remained significant (adjusted OR = 1.290, p = 0.044, 95% CI 1.006, 1.654) after adjusting for diabetes, diabetic medications and FBG. The association between FBG and statin use was not significant after adjustment.

**Table 2:** Profiles of hypertensive patients who were statins users versus non-users

	**Statin users (n = 810)**	**Statin non users (n = 250)**	**P-value**	**95% CI**
Mean (SD) age (years)	62.23 (9.81)	61.16 (12.16)	0.205*	-2.725, 0.586
DM [n(%)]	415 (81.9)	92 (18.1)	<0.001**	
Non DM [n(%)]	395 (71.4)	158 (28.6)		
On DM medication [n(%)]	391 (83.7)	76 (16.3)	<0.001**	
Not on DM medication [n(%)]	419 (70.7)	174 (29.3)		
HbA1c#	n = 444	n = 110		
Mean IFCC unit (SD) mmol/mol	59 (19)	52 (16)	0.001*	-10.378, -2.801
Mean NGSP unit (SD) %	7.5 (1.7)	6.9 (1.5)	0.001*	-0.942, -0.247
FBG#	n = 746	n = 219		
Mean (SD) mmol/L	6.63 (2.49)	6.05 (1.93)	<0.001*	-0.886, -0.261

DM=Diabetes mellitus; HbA1c=Glycated haemoglobin; FBG=Fasting blood glucose.

IFCC = International Federation of Clinical Chemistry; NGSP = National Glycohemoglobin Standardization Program.

#Most recent result in the past one year.

*Independent sample *t*-test.

**Chi-square test.

Table 3 shows the results of the analysis on two subgroups of the study population: 1) hypertensive patients with diabetes, and 2) hypertensive patients without diabetes. In the group of hypertensive patients with diabetes, statin users had significantly higher HbA1c levels compared to statin non-users. This was despite the significantly higher use of diabetic medications in the statin user group. Among the hypertensive patients with diabetes, 92.1% were on diabetic medications. The diabetic medications taken were metformin (n = 422, 83.2%), sulphonylurea (n = 296, 58.4%), acarbose (n = 53, 10.5%), gliptins (n = 2, 0.4%) and insulin (n = 75, 14.8%). The difference in HbA1c levels remained significant after adjustment for age and diabetic medication (adjusted OR 1.208, p = 0.037, 95% CI 1.012, 1.441). There was no difference in the FBG level, HDL levels, age, BMI, gender, ethnicity, diuretics and beta blockers use, and duration of diabetes between statins users and non-users in the study population who had diabetes. Triglyceride levels were significantly lower in statin users compared to non-users in those with diabetes.

**Table 3:** Comparison of socio-demographic factors and glucose control of hypertensive patients with and without diabetes who were statins users or non-users

	**Hypertensive patients with DM (n = 507)**			**Hypertensive patients without DM (n = 553)**		
	**Statin users**	**Statin non users**	**P value (95% CI)**	**Statin users**	**Statin non users**	**P value (95% CI)**
	**n = 415**	**n = 92**		**n = 396**	**n = 157**	
Age (years) Mean (SD)	61.71 (9.29)	62.17 (12.02)	0.728* (-2.176, 3.107)	62.80 (10.30)	60.52 (12.23)	0.039* (-4.463, -0.111)
Gender n (%)						
Men	176 (84.6)	32 (15.4)	0.178**	155 (70.8)	64 (29.2)	0.725**
Women	239 (79.9)	60 (20.1)		241 (72.2)	93 (27.8)	
Ethnicity n (%)						
Malay	137 (83.5)	27 (16.5)	0.737**	116 (73.0)	43 (27.0)	0.808**
Chinese	160 (80.4)	39 (19.6)		220 (71.0)	90 (29.0)	
Indian	115 (81.6)	26 (18.4)		54 (70.1)	23 (29.9)	
Others	3 (100.0)	0 (0.0)		6 (85.7)	1 (14.3)	
Duration of diabetes (years)	n = 406	n = 84				
Mean (SD)	8.169 (6.556)	7.020 (6.255)	0.141*			
HbA1c#	n = 372	n = 77		n = 72	n = 33	
Mean IFCC unit (SD) mmol/mol	62 (19)	57 (17)	0.030* (-9.610, -0.494)	44 (5)	42 (6)	0.082* (-3.972, 0.242)
Mean NGSP unit (SD)%	7.8 (1.7)	7.3 (1.6)	0.034* (-0.871, -0.034)	6.1 (0.4)	6.0 (0.5)	0.089* (-0.363, 0.026)
FBG#	n = 397	n = 87		n = 350	n = 131	
Mean (SD) mmol/L	7.71 (2.98)	7.25 (2.55)	0.179* (-1.142, 0.213)	5.39 (0.53)	5.27 (0.58)	0.029* (-0.231, -0.012)
TG#	n = 383	n = 84		n = 361	n = 137	
Mean (SD) mmol/L	1.602 (0.737)	1.965 (1.289)	0.014* (0.075, 0.653)	1.416 (0.623)	1.357 (0.628)	0.346* (-0.182, 0.064)
HDL#	n = 383	n = 84		n = 361	n = 137	
Mean (SD) mmol/L	1.269 (0.317)	1.204 (0.322)	0.092* (-0.140, 0.011)	1.465 (0.420)	1.373 (0.380)	0.026* (-0.173,-0.011)
Body Mass Index	n = 232	n = 44		n = 150	n = 58	
Mean (SD) Kg/m^2^	28.02 (5.37)	27.74 (4.70)	0.744* (-1.990, 1.423)	27.44 (4.67)	26.73 (5.35)	0.350* (-2.189, 0.778)
Diabetic medication n (%)						
Yes	391 (83.7)	76 (16.3)	<0.001**			
No	24 (60.0)	16 (40.0)				
On diuretic and/or beta-blocker n (%)						
Yes	201 (80.1)	50 (19.9)	0.305**	196 (74.5)	67 (25.5)	0.148**
No	214 (83.6)	42 (16.4)		200 (69.0)	90 (31.0)	

DM = Diabetes mellitus; HbA1c = Glycated haemoglobin; FBG = Fasting blood glucose; TG = Triglyceride; HDL = HDL-Cholesterol.

IFCC = International Federation of Clinical Chemistry; NGSP = National Glycohemoglobin Standardization Program.

# Most recent result in the past one year.

*Independent sample *t*-test.

**Chi-square test.

In the group of hypertensive patients without diabetes, age and FBG values were significantly higher among statin users compared with non-users. However, after adjustment for age and FBG, both factors became non-significant. There was no significant association found between HbA1c levels and statin use. The number of patients with HbA1c results was small in this group (Table 3).

## Discussion

There are two key findings from this study – 1) patients with hypertension and those with coexisting diabetes who used statins have significantly higher HbA1c levels compared to those not using statins and 2) the poor glycaemic control is not reflected by fasting glucose levels.

Higher HbA1c levels in statin users for the overall study population of hypertensive patients with and without diabetes suggests that statins affect glycaemic control not just in patients with diabetes but in hypertensive patients without diabetes as well. The association between HbA1c and statin remained significant after adjustment for possible confounders. Although this association was not significant in subgroup analysis for the non-diabetics, this could possibly be due to the small number of patients with HbA1c results in this subgroup. There was a similar trend for fasting blood glucose levels in non-diabetics but this was non-significant after adjustment for confounders. Poor glycaemic control with statin use could increase cardiovascular risk in the long-term in patients with hypertension on statins. Therefore regular monitoring of glycaemic control is important in this group of patients.

Fasting blood glucose level was higher in patients with diabetes who were on statins, but this did not reach significance. A significantly greater proportion of diabetics on statins were prescribed diabetic medications compared with diabetics not on statins. It is a possibility that poorer glycaemic control in the diabetics on statin group necessitates the use of medication i.e. improving fasting blood glucose control but not improving overall glycaemic control as reflected by the higher HbA1c levels.

Antihypertensive medications such as diuretics and beta blockers as well as diabetes duration could potentially affect glycaemic control. However, the analysis showed that these antihypertensive agents and the duration of diabetes were not significantly associated with statin use in patients with diabetes. As expected, triglyceride levels were significantly lower in statin users compared to non-users in those with diabetes.

Most of the previous studies which have shown similar findings were conducted in clinical trial participants, which had strict controlled settings. This is a retrospective cohort, which could only show association and not causation. It is possible that the association seen between statins and hyperglycaemia may be due to the clustering of hyperlipidaemia, hypertension and glucose intolerance, which are known features of metabolic syndrome. Nevertheless, the association was also seen in patients who did not have diabetes although this was not significant after adjustment. However, it has highlighted that statin is associated with poorer glycaemic control in patients with diabetes in a ‘real-life’ setting. Doctors may not have recognised this previously because the fasting blood glucose levels do not increase significantly and the effect is lessened by medication. Although there appears to be a response by prescribers to the poorer glycaemic control in that the number of diabetes medications was increased in this group, yet this response was insufficient to control the higher HbA1c levels.

The findings from this observational cohort concur with trials which indicate that statins are associated with poorer glycaemic control in patients with diabetes and hypertension. Statins have been shown to affect glucose metabolism in multiple ways such as by inhibition of insulin secretion and downregulation of a glucose transporter in adipocytes [12]. Clinicians managing patients with diabetes should take note of this and ensure that glycaemic control is managed accordingly.

## Conclusions

Statin use is associated with increased HbA1c levels among hypertensive patients and hypertensive patients with diabetes. As the evidence for statin therapy in reducing mortality is much stronger than the evidence to support glucose lowering therapy, clinicians managing hypertensive patients with diabetes on statins should not stop statin use but ensure that hyperglycaemia is kept under control. For patients with hypertension and using statins, regular HbA1c monitoring may be useful.

## Abbreviations

BMI: Body mass index; DM: Diabetes mellitus; FDA: Food and Drug Administration; FBG: Fasting blood glucose; HbA1c: Glycated haemoglobin; IFCC: International Federation of Clinical Chemistry; NGSP: National Glycohemoglobin Standardization Program; UKPDS: United Kingdom Prospective Diabetes Study.

## Competing interests

The authors declare that they have no competing interests.

## Authors’ contribution

SM, PY and EM drafted the manuscript. All authors participated in the conception and design of the study. SM, PY, NF and EM performed the statistical analysis. All authors read and approved the final manuscript.
